# Ocular Motility Patterns in Intellectual Disability: Insights from the Developmental Eye Movement Test

**DOI:** 10.3390/life13122360

**Published:** 2023-12-18

**Authors:** Elvira Orduna-Hospital, Diego Hernández-Aranda, Ana Sanchez-Cano

**Affiliations:** Department of Applied Physics, University of Zaragoza, 50009 Zaragoza, Spain; 805549@unizar.es

**Keywords:** developmental eye movement test, eye tracker, intellectual disability, ocular motility

## Abstract

Purpose: To measure the ocular motility parameters of the Developmental Eye Movement (DEM) test objectively, with an eye tracker in subjects with intellectual disability (ID). Methods: The DEM test was performed on 45 subjects with ID, while their eye movements were recorded with an eye tracker. Some objective parameters of ocular motility were obtained through each subtest (A, B, and C) of the full DEM test. Results: There was a significant positive correlation between the saccadic speed (cc: 0.537; *p* = 0.001) and length (cc: 0.368; *p* = 0.030) of both eyes for the same subject. People with a higher percentage of ID exhibited a greater number of fixations, saccades, and errors, and took longer to perform the DEM test than those with a lower ID percentage, who had greater numbers of these parameters than subjects without ID. Subjects without ID exhibited faster saccades, with a higher amplitude, than subjects with ID. Conclusions: The eye tracker quantifies ocular motility parameters involved in the DEM test in subjects with ID. Both eyes’ movements in subjects with ID were conjugated, exhibiting saccades of the same length and speed. All parameters were different in subjects with ID compared to those in subjects without ID, so normative tables specifically for subjects with ID are necessary.

## 1. Introduction

Intellectual disability (ID) is characterized by a slow and incomplete development of human cognitive abilities that contribute to the level of general intelligence, as well as to motor, social, and language abilities. In addition, individuals with ID have a reduced ability to understand, learn, remember new things, and even communicate [1,2]. The causal or associated factors of ID can be organic, genetic, or sociocultural, although there is no one specific factor, as most cases involve a combination of factors. Trisomy of chromosome 21 (Down syndrome) and fragile X syndrome are the most common genetic causes of ID [2]. Disability presents before the age of 18, and its prevalence is estimated to range from 1% to 3% worldwide, with a higher occurrence in males [2]. Individuals with ID typically have an intelligence quotient (IQ) below 70–75. However, the diagnosis of ID is not solely based on IQ, as other factors, such as social adaptation and daily functioning, are also taken into account [3].

ID can vary in degree and severity, and individuals with ID may have different abilities and needs. Royal Decree (RD) 888/2022 of Spain, which aligns with the social model of the United Nations Convention on the Rights of Persons with Disabilities, came into effect on 20 April 2023 [4]. With this new legislation, the following levels of ID were established [5]: Grade 0: 0–4%, the person does not face difficulties in carrying out daily activities; Grade 1: mild (5–24%), with which the person is independent in almost all daily activities; Grade 2: moderate (25–49%), with which the person has independence in self-care but faces some difficulties in daily activities; Grade 3: severe (50–95%), with which the person experiences high difficulty in performing daily activities, including self-care; and Grade 4: profound or complete (96–100%), with which the person has absolute difficulty in daily activities and self-care.

Motor limitations result in imprecise eye movements that affect the vision and learning of subjects with ID, manifesting most commonly in reading. In addition, individuals with ID tend to have more visual problems than people without ID, including refractive errors, strabismus, cataracts, keratoconus, nystagmus, and binocular dysfunctions. Therefore, better optical and visual quality would be useful in the development of skills such as learning and integration, resulting in a better quality of life [1].

During the reading process, the eyes perform fixation eye movements to extract and process visual information, accompanied by saccadic movements that have different amplitudes depending on the spacing between words, following the direction marked by the lines of the text [6,7,8]. Uncontrolled movements occur with ocular motility problems, making the reading process difficult and diminishing the comprehension of the text [9].

The Developmental Eye Movement (DEM) test is a psychometric assessment used to evaluate oculomotor performance [10]; it simulates a reading environment to assess and quantify oculomotor abilities during reading, particularly saccadic eye movements, facilitated by visuo-verbal skills [11]. The DEM test consists of a preliminary or pretest and three additional subtests: two vertical reading subtests (subtest A and subtest B) and one horizontal reading subtest (subtest C) [10]. The test utilizes a correction template and standardized parameter tables based on age, as our group described in a previous research paper [12]. The vertical time (VT), adjusted horizontal time (AdjHT), ratio, and error count are measured and referenced against age-normalized tables to determine percentiles, which indicate whether the subject’s performance is within a normal, high, or low percentile range. A percentile equal to or greater than the 31st percentile is considered normal [10]. To our knowledge, this test has not been used in a study of subjects with ID. Given the strong association between the DEM test and reading ability, it can be useful in identifying individuals with reading delays [11]. It is also valuable for assessing children with learning difficulties [10] and, in the case of this study, individuals with ID. Based on the analysis of previous results, four different categories can be determined: Type I, in which the AdjHT, VT, ratio, and errors are normal for the subject’s age; Type II, in which the VT is normal and the AdjHT is higher, resulting in a higher ratio, indicating potential issues with horizontal saccadic movements; Type III, in which both the VT and AdjHT are higher than normal for the subject’s age, but the ratio is normal, indicating difficulties with automaticity in counting numbers; and Type IV, a combination of Type II and Type III, in which the AdjHT, VT, and ratio are all elevated, indicating problems with both automaticity and oculomotor skills [13].

The eye tracker is an ocular tracking system that uses near-invisible infrared readers and high-definition cameras; it is capable of identifying and analyzing fixation stability and saccadic movement precision, determining where a subject is looking while performing the investigated tasks [7,12]. The data provided with the eye tracker can be considered unbiased, objective, and quantifiable, since the subject’s ocular movements can be observed in real time on the computer screen [12]. Once analyzed, fixations and saccades can provide clues about the subject’s motivations or cognitive processing. Additionally, the cognitive processes involved in reading can be studied based on the duration of fixations and saccades, which are longer in more challenging parts of the text or due to greater word ambiguity [7].

The main objective of this study was to quantitatively assess the eye movements made by people with ID during the DEM test using an eye tracker and to calculate the percentiles of the DEM test for a sample of subjects with ID. The eye tracker was used to try to obtain objective data about the execution time of each subtest and the errors made when performing it, and to quantify the fixations and saccades, as well as the speed and amplitude of the latter, in a group of subjects with different degrees of ID. In addition, this study aimed to compare the results of subtest C in subjects with ID against subjects without ID.

## 2. Materials and Methods

### 2.1. Sample Description and Selection

The Clinical Research Ethics Committee of Aragón (CEICA) (reference number PI21-074) approved this research, which was conducted from January to April 2023, with the collaboration of the subjects with ID, who attend the Tasubinsa Center in Navarra (Spain) on a daily basis. All individuals within the age range of 18–40 years who agreed to participate were eligible. The objectives of the study were explained, and detailed information was provided about the tests that would be performed. The participants and, in some cases, their legal guardians, signed the informed consent forms.

A screening was performed to identify subjects who met the following inclusion criteria: spherical refractive error between −6.00 D and +3.00 D and astigmatic error of less than 3.00 D; no systemic or ocular pathologies; visual acuity (VA) equal to or greater than 0.80 decimal (20/25 Snellen); and no suppression of one of the eyes, strabismus, or amblyopia.

### 2.2. Exploratory Protocol

Each subject’s habitual optical correction was measured using a lensometer, VA with correction was assessed, and phoria was quantitatively evaluated using the Maddox Rod test, a light stimulus, and a prism bar. These analyses were performed for distance and near vision, and vertical and horizontal phorias were measured. Participants who met the inclusion criteria had their ocular motility assessed using the DEM test monitored with an eye tracker (Tobii Pro Fusión, Tobii AB, Danderyd, Sweden).

The DEM test was digitized and calibrated for a 21-inch screen, connected to the laptop from which the eye tracker was controlled, and voice was recorded.

The participants were positioned with their chin and forehead resting on the chin rest, 60 cm from the screen, with the DEM test calibrated for a VA of 0.8. The eye tracker was situated just below the screen, also at a distance of 60 cm from the participants.

Once the DEM test was explained, the subjects performed the 4 DEM subtests (pretest, subtest A, subtest B, and subtest C) individually, reading the numbers aloud for the recording of errors (substitutions, additions, omissions, and transpositions). The time it took to perform each subtest was obtained from the eye tracker recordings.

### 2.3. Data Collection

After the DEM test was performed on all of the subjects, the Tobii Pro Lab program (Tobii AB, Danderyd, Sweden) was used to confirm that the eye tracker had captured both eyes, and the recordings were segmented into four “events”, one for each subtest performed. The duration of these cuts was determined by the first and last fixation that the subject made during each of the subtests.

The recordings of each subject were transferred to the EyeTracker Parse program (University of Zaragoza, Zaragoza, Spain). This program selected the variables from each event, such as the duration of each subtest (s), the number of fixations and saccades in each subtest (n), the pupillary diameter of both the right eye (RE) and the left eye (LE) (mm), and the saccadic speed (m/s) and length (mm) for each eye. Then, each subtest from each participant was analyzed separately and exported to Excel (Microsoft^®^ Office Excel 2011, Microsoft Corporation, Redmond, WA, USA) to create one Excel database for each subtest (subtest A, subtest B, and subtest C).

### 2.4. Statistical Analysis

The variables were analyzed with the Statistical Package for the Social Sciences (SPSS 24.0 Inc., Chicago, IL, USA). The mean, standard deviation, and maximum and minimum values were calculated for each parameter. The sample did not follow normality according to the Kolmogorov–Smirnov test, so the nonparametric Wilcoxon signed rank test was performed for related samples, and the correlation was determined with Spearman’s test. Variables of subtest C were also compared between subjects with ID and subjects without ID, for which the nonparametric Mann–Whitney U test for independent samples was used. A *p* value < 0.05 was considered statistically significant. Percentiles, scatter plots, and bar plots were generated using Excel.

## 3. Results

### 3.1. Results of Subjects with ID

In total, 45 subjects, comprising 24 men and 21 women, between 22 and 40 years old with a mean age of 31.15 ± 4.26 years, were included. The percentage of ID varied from 33% to 76%, with a mean of 55.13% ± 12.76%.

Table 1 shows the results obtained in each of the subtests. The number of fixations was lower than the number of saccades among the three subtests. The saccadic speed was slightly lower in the RE (subtest A: 0.88 ± 0.36 s, subtest B: 0.82 ± 0.38 s, subtest C: 1.04 ± 0.44 s) than in the LE (subtest A: 1.06 ± 0.57 s, subtest B: 1.02 ± 0.52 s, and subtest C: 1.11 ± 0.45 s). In turn, the saccadic length was also lower in the RE (subtest A: 32.75 ± 20.97 s, subtest B: 27.16 ± 19.38 s, and subtest C: 29.91 ± 11.72 s) than in the LE (subtest A: 40.22 ± 25.02 s, subtest B: 38.99 ± 21.57 s, and subtest C: 38.18 ± 18.90 s).

The VT (57.81 ± 21.93 s), AdjHT (81.68 ± 35.96 s), ratio (1.50 ± 0.59), and number of errors (5.50 ± 8.69) were calculated for the ID group (Table 2).

The mean VT was in the 35th percentile, the mean AdjHT was in the 40th percentile, and the mean ratio was in the 35th percentile (Table 2).

**Figure 1:**
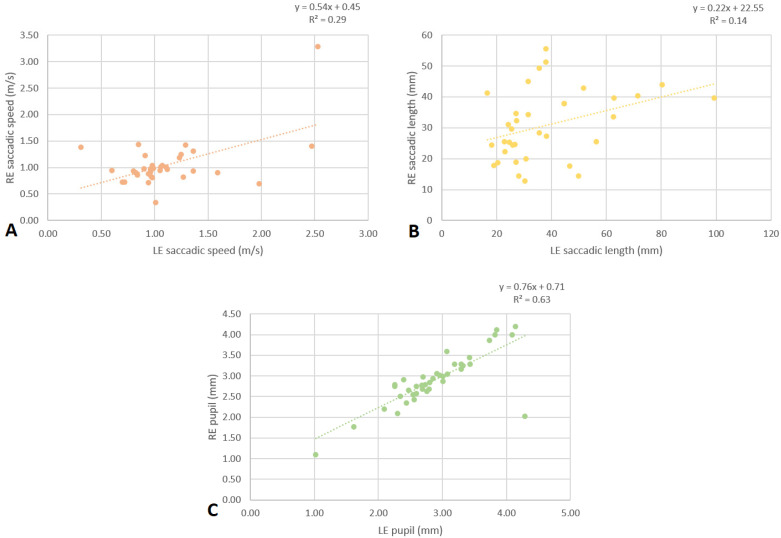
Scatter plots with regression lines and equations correlating the speed (**A**) and length (**B**) of RE and LE saccades and the RE pupil size with the LE pupil size (**C**) in subjects with ID (*n* = 45).

There was a significant positive correlation between the saccadic speed (cc: 0.537; *p* = 0.001) and the saccadic length (cc: 0.368; *p* = 0.030) of the RE and the LE, as well as a positive correlation between the pupil size of both eyes (cc: 0.793; *p* < 0.001), for subjects with ID (*n* = 45) in subtest C (Figure 1).

### 3.2. Results Obtained by Dividing the Subjects with ID into Two Groups According to Their Degree of ID

The subjects were divided into two groups, according to the degree of ID they possessed. Group 1 comprised 25 subjects, with a disability percentage between 33% and 64%, and Group 2 comprised 20 subjects, with an ID percentage between 65% and 76%.

The mean age of Group 1, with 10 men and 15 women, was 31.73 ± 3.82 years (24–39 years), and the mean percentage of disability was 45.48 ± 8.26% (33–64%). In Group 2, with 14 men and 6 women, the mean age was 30.42 ± 4.76 years (22–38 years), and the mean percentage of ID was 67.20 ± 3.89% (65–76%).

The subtest C duration in Group 2 was statistically greater than that of Group 1 (Group 1: 63.58 ± 19.80 s, Group 2: 88.40 ± 27.74 s; *p* = 0.008). Group 2 performed a greater number of fixations (Group 1: 159.54 ± 49.08, Group 2: 193.94 ± 52.85; *p* = 0.040) and saccades (Group 1: 259.25 ± 142.61, Group 2: 585.56 ± 577.57; *p* = 0.037) than Group 1, and both differences were statistically significant (Table 1). The saccadic speed was faster in Group 1 (RE: 1.10 ± 0.54 m/s, LE: 1.12 ± 0.45 m/s) than in Group 2 (RE: 0.97 ± 0.27 m/s, LE: 1.10 ± 0.47 m/s), but there were no significant differences between groups for either eye (RE: *p* = 0.591 and LE: *p* = 0.987). No statistically significant differences were found for saccadic length, but the RE length was longer in Group 2 than in Group 1 (Group 1: 28.03 ± 11.37 mm, Group 2: 32.39 ± 12.07 mm; *p* = 0.290), while the LE length was greater in Group 1 than in Group 2 (Group 1: 38.79 ± 19.32 mm, Group 2: 37.45 ± 19.00 mm; *p* = 0.679) (Table 1).

The VT, AdjHT, ratio, and number of errors were calculated for each group, as shown in Table 3. The VT of Group 1 was lower than that of Group 2 (Group 1: 47.04 ± 10.48 s, Group 2: 73.37 ± 24.92 s), and the AdjHT was also shorter in Group 1 than in Group 2 (Group 1: 71.78 ± 32.11 s, Group 2: 98.18 ± 36.99 s). The ratio was higher for Group 1 than for Group 2 (Group 1: 1.55 ± 0.69, Group 2: 1.40 ± 0.38). The subjects in Group 2 made more errors when performing subtest C than those in Group 1 (Group 1: 4.92 ± 9.24, Group 2: 6.47 ± 7.88).

### 3.3. Differences between People with ID and People without ID

The subtest C results, shown above, for the subjects with ID were compared with those of a sample of subjects without ID. The inclusion criteria for this control group were the same as for the group with ID, aside from the fact that they did not suffer from ID. This sample without ID included 52 subjects, of which 30 were women and 22 were men, with a mean age of 21.00 ± 3.22 years (from 18 to 30 years). This group also signed informed consent forms to participate.

The mean VA of the group without ID was −0.04 ± 0.06 LogMAR, and that for the group with ID was 0.02 ± 0.09 LogMAR. No differences were found in VA between groups (*p* = 0.182).

**Figure 2:**
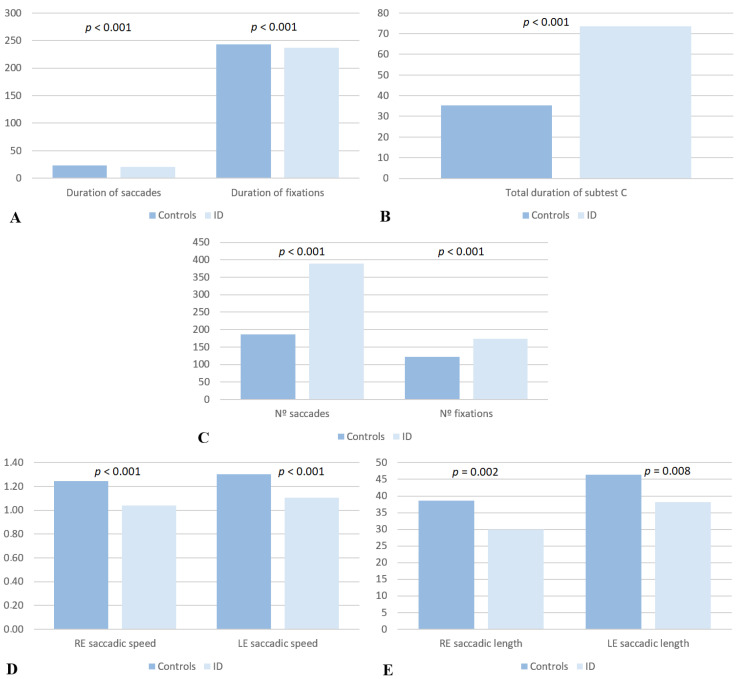
Comparative graph of the saccade and fixation durations (s) (**A**), the total duration (s) of subtest C (**B**), the number of saccades and fixations (**C**), and the saccadic speed (m/s) (**D**) and length (mm) (**E**) of the subjects without ID (*n* = 52) and the subjects with ID (*n* = 45). The differences between groups, with a *p* value < 0.05, was considered statistically significant.

Both saccades and fixations showed significant differences, with a shorter duration in subjects without ID than in subjects with ID (Figure 2A). In Table 1 and Figure 2B, it can be seen that the subjects without ID had a significantly shorter duration for subtest C than the subjects with ID (subjects without ID: 35.25 ± 6.68 s, subjects with ID: 73.51 ± 26.06 s; *p* < 0.001). In addition, Figure 2C shows that the subjects without ID performed fewer fixations (subjects without ID: 121.14 ± 15.24, subjects with ID: 173.30 ± 52.78; *p* < 0.001) and saccades (subjects without ID: 186.66 ± 96.87, subjects with ID: 389.78 ± 408.05; *p* < 0.001) than the subjects with ID, with significant differences in both cases.

Saccades (Figure 2D) were significantly faster in subjects without ID (RE: 1.25 ± 0.38 m/s, LE: 1.30 ± 0.36 m/s) than in subjects with ID (RE: 1.04 ± 0.44 m/s, LE: 1.11 ± 0.45 m/s). The saccadic length (Figure 2E) was significantly longer in subjects without ID (OD: 38.56 ± 12.41 mm, LE: 46.42 ± 20.47 mm) than in subjects with ID (OD: 29.91 ± 11.72 mm, LE: 38.18 ± 18.90 mm) (Table 1).

The subjects without ID exhibited a lower VT and AdjHT (VT: 33.58 ± 5.56 s, AdjHT: 35.24 ± 6.68 s) than the subjects with ID (VT: 57.81 ± 21.93 s, AdjHT: 81.68 ± 35.96 s). The ratio was also lower in subjects without ID (1.05 ± 0.09) than in subjects with ID (1.50 ± 0.59). The subjects without ID did not make any errors (since it was an inclusion criterion), while the subjects with ID had a mean of 5.50 ± 8.69 errors (Table 3).

## 4. Discussion

This study aimed to objectively evaluate the ocular motility parameters of the DEM test using an eye tracker in subjects with ID. The DEM test is a visual–verbal test, in which movements such as saccades or fixations, involved in seeing, recognizing, and naming numbers, are measured. Observing the results of this study in participants with ID, there was a significant positive correlation between the length and speed of the saccades of the RE and those of the LE, as well as the pupillary diameter; these results are consistent with those found in a similar study in subjects without ID [12], which indicates that both eyes of the same subject work together even with ID.

When analyzing the mean traditional parameters of the DEM test in subjects with ID, the VT was in the 35th percentile, the AdjHT was in the 40th percentile, and the ratio was in the 35th percentile. These parameters would be considered normal for the ID group, since they are above the 31st [10] percentile according to the percentiles of their own ID group (Table 2). However, according to the normalized percentile table for a sample of subjects without ID, the VT, AdjHT, and ratio values would be in the 1st percentile [12]. With these results, it could be concluded that people with ID take longer to perform the different subtests and exhibit worse comprehension, since the number of mean errors was 5.50.

Because subjects with ID tend to have more oculomotor dysfunctions, such as nystagmus, abnormal values are expected and can serve as a reference for other investigations. We tried to obtain percentiles for the subjects with ID and determine if there was any difference in the results depending on the degree of ID. When the ID group was divided into two subgroups based on the percentage of ID, the VT of Group 1 was 47.04 s, while that of Group 2 was significantly higher (*p* = 0.008), at 73.37 s. The AdjHT was 71.78 s for Group 1 and 98.18 s for Group 2. The ratio was higher for Group 1 than for Group 2 (1.55 and 1.40, respectively). The subjects in Group 2 (6.47) made a greater number of errors than those in Group 1 (4.92). The subjects in Group 2 exhibited a greater number of interruptions and had more difficulty understanding the dynamics of the test than those in Group 1. In addition, Group 1 exhibited a higher percentile (60) in VT than Group 2 (15). For the AdjHT, Group 1 exhibited the 45th percentile, while Group 2 exhibited the 20th percentile. However, for the ratio, Group 1 exhibited the 30th percentile, and Group 2 exhibited the 50th percentile, in some cases similar to a Type IV DEM test result [13]. When performing the test, the explanation had to be repeated several times for some subjects to complete the test in its entirety. Subjects with ID also took longer to understand the format of subtest C than the formats of subtests A and B.

All subjects with ID were compared with a sample of 52 subjects without ID. This comparison verified that the subjects with ID had a greater VT (57.81 ± 21.93 s) than the subjects without ID (33.58 ± 5.56 s), and the same pattern was observed for the AdjHT (subjects with ID: 81.68 ± 35.96 s, subjects without ID: 35.24 ± 6.68 s). In addition, the number of errors made by the subjects with ID was 5.50 ± 8.69, while the subjects without ID did not make any errors. The ratio in the subjects with ID was 1.50 ± 0.59, while in the subjects without ID, it was 1.05 ± 0.09.

This study standardized the DEM test in subjects with ID. To achieve this, we evaluated 45 subjects between the ages of 22 and 40 with a mean age of 31.15 ± 4.26 years. It also demonstrates that it is necessary to create normalized tables with different percentiles for subjects with ID than those used for people without ID, since their fixations and saccades are slower and more numerous, which does not necessarily mean that they have motility problems, but that they are typical for their percentage of ID. In other words, both eyes of the same subject with ID work together, without differences between them, and with a significant positive correlation in terms of saccadic speed and length.

Scheiman [14] postulated that the fewer saccades performed and the more parafoveal vision is used, the faster the reading, and the better the comprehension will be [15]. On the other hand, when analyzing a study in which different images were presented to children with mild ID and children without ID, monitored with a GP3 Gazepoint eye-tracking system (Vancouver, BC, Canada), children with mild ID performed fewer fixations than children without ID. The opposite occurred in our study, in which subjects with ID performed a greater number of fixations than subjects without ID. The previous study [14] suggested that children with mild ID make fewer fixations because they need more time to carry them out. Furthermore, the children with mild ID ignored textual elements in mixed images, instead focusing their attention on the pictorial elements, while the children without ID fixed their gaze more on the textual elements. Therefore, the DEM test may seem unattractive to subjects with ID and take longer to complete. However, subjects with a high percentage of ID were made to look at the numbers and name them, which is generally more difficult than for letters, so fixations are maintained longer.

Other studies have been performed in diverse populations, using devices to monitor ocular motility as objectively as possible. Vakil et al. [16] compared subjects with ID to subjects with typical development (TD), matched by cognitive level. Participants solved perceptual and conceptual analogies (from the Conceptual and Perceptual Analog Modifiability Test, CPAM), while their eye movements were monitored with an eye tracker, with the TD group having a significantly higher overall percentage of correct answers. The eye movement pattern revealed that there were both quantitative and qualitative differences between the groups in the process of solving the analogies. The authors interpreted this as a reflection of two different types of strategies: constructive matching and response elimination.

In another investigation [17], the reaction times of saccades were studied in subjects with ID between 13 and 57 years old, using electrooculography, taking into account the IQs of the subjects. It was found that subjects with a higher IQ exhibited a shorter mean saccade reaction time. Although the methodology was different, these conclusions could be reflected in our study, in that subjects with a higher percentage of ID had a longer test duration, since their saccades need a longer reaction time.

In our study, subjects with ID made a greater number of errors on subtest C than on subtests A or B, possibly caused by reading the same line twice or skipping to the next line, since test C is a multiline task [18]. Therefore, as Hindmarsh et al. [7] commented, both horizontal and vertical eye movements must be considered to understand the behavior of eye movements during reading.

Conducting a study, with an eye tracker, on the duration of fixations and saccades, as well as the saccadic length and speed of each eye of the same subject with ID, could be useful to detect subjects with eye movement conjugation problems that can affect their reading. This procedure cannot be used as a diagnostic tool for ID, but it can be very useful to understand potential deficiencies in reading. For this reason, it is important to evaluate the DEM test results with normative tables for subjects with ID with their own percentiles, since using normative tables for subjects without ID will result in very low percentiles in the absence of ocular motility dysfunction, due to the intrinsic effects of having an ID.

Individuals with varying ID severities may exhibit distinctive ocular motility features. Those with higher ID percentages, indicative of more severe ID, may struggle to coordinate eye movements during the DEM test, thus impacting both saccadic speed and accuracy. Factors like increased fixation counts and prolonged trial times, particularly in individuals with higher ID percentages, add complexity to interpreting ocular motility data. This necessitates normative tables tailored for those with ID, acknowledging potential deviations from patterns observed in individuals without ID. In clinical evaluations of people with ID, it is crucial to consider the multifaceted nature of ID when interpreting ocular motility results. This study’s establishment of a 76% disability limit underscores the challenges faced by those with higher ID percentages, highlighting slower and less precise eye movements in Group 2 individuals.

A future line of research in subjects with ID could be to perform the test for adults with two-digit numbers, using the Adult Developmental Eye Movement (ADEM) test [19], or its modified version, the ADEMd test [20], which involves a task that requires more attention. It could offer valuable insights into the nuanced aspects of ocular motility in people with ID.

## 5. Conclusions

The eye tracker is an objective method of evaluating the DEM test in that can quantify the ocular motility parameters in subjects with ID, not determined using the traditional subjective method. The eye movements of both eyes for subjects with ID were conjugated, exhibiting saccadic movements of the same length and speed. All parameters were different in subjects with ID compared to subjects without ID, so normative tables specifically for subjects with ID are necessary.

## Figures and Tables

**Table 1:** Means, standard deviations, and minimum and maximum (in brackets) of the data obtained from the evaluation of the DEM test with eye tracker in all people with ID (*n* = 45), divided into two groups according to the percentage of ID (subjects with ID were divided into two groups according to their ID degree: Group 1 comprised 25 subjects with a disability between 33% and 64%, and Group 2 comprised 20 subjects with an ID between 65% and 76%), and the group without ID (*n* = 52). The differences between groups, with a *p* value < 0.05, was considered statistically significant and is marked in bold. RE, right eye; LE, left eye.

	People with ID (*n* = 45)			Group 1 (*n* = 25)	Group 2 (*n* = 20)	People without ID (*n* = 52)	Group 1 vs. Group 2	People with ID vs. People without ID
	Subtest A	Subtest B	Subtest C				*p* Value	
Duration (s)	28.13 ± 10.67 [13.70–59.93]	29.68 ± 11.51 [14.77–66.13]	73.51 ± 26.06 [34.92–15.28]	63.58 ± 19.80 [34.92–114.53]	88.40 ± 27.74 [53.87–152.78]	35.24 ± 6.68 [25.79–49.71]	**0.008**	**<0.001**
Number of fixations (n)	46.64 ± 31.27 [13–159]	50.30 ± 29.52 [11–140]	173.30 ± 52.78 [34–304]	159.54 ± 49.08 [34–265]	193.94 ± 52.85 [111–304]	121.14 ± 15.24 [91–167]	**0.040**	**<0.001**
Number of saccades (n)	121.64 ± 167.24 [14–830]	141.89 ± 186.72 [14–788]	389.78 ± 408.05 [104–2230]	259.25 ± 142.61 [104–579]	585.56 ± 577.57 [149–2230]	186.66 ±96.87 [101–674]	**0.037**	**<0.001**
RE saccadic speed (m/s)	0.88 ± 0.36 [0.32–1.76]	0.82 ± 0.38 [0.36–2.67]	1.04 ± 0.44 [0.34–3.28]	1.10 ± 0.54 [0.69–3.28]	0.97 ± 0.27 [0.34–1.42]	1.25 ± 0.38 [0.21–2.89]	0.591	**<0.001**
LE saccadic speed (m/s)	1.06 ± 0.57 [0.56–3.10]	1.02 ± 0.52 [0.33–2.83]	1.11 ± 0.45 [0.31–2.53]	1.12 ± 0.45 [0.60–2.53]	1.10 ± 0.47 [0.31–2.47]	1.30 ± 0.36 [0.71–3.06]	0.987	**<0.001**
RE saccadic length (mm)	32.75 ± 20.97 [6.53–85.00]	27.16 ± 19.38 [6.54–70.60]	29.91 ± 11.72 [11.21–55.56]	28.03 ± 11.37 [11.21–51.35]	32.39 ± 12.07 [14.36–55.56]	38.56 ± 12.41 [15.39–92.39]	0.290	**0.002**
LE saccadic length (mm)	40.22 ± 25.02 [9.94–114.27]	38.99 ± 21.57 [10.75–146.08]	38.18 ± 18.90 [16.68–99.31]	38.79 ± 19.32 [18.19–99.31]	37.45 ± 19.00 [16.68–80.31]	46.42 ± 20.47 [22.13–148.14]	0.679	**0.008**

**Table 2:** Means and standard deviations (±SD) of the vertical time (VT), adjusted horizontal time (AdjHT), ratio, number of errors, and DEM test percentiles for a group of 45 subjects with ID.

*n* = 45	VT (s)	AdjHT (s)	Ratio	Errors
Mean ± SD	57.81 ± 21.93	81.68 ± 35.96	1.50 ± 0.59	5.50 ± 8.69
Percentiles				
P99	30.99	37.09	1.02	
P95	35.67	40.62	1.06	
P90	38.26	43.04	1.08	
P85	40.08	49.00	1.09	
P80	41.41	54.71	1.10	
P75	42.53	56.24	1.13	
P70	43.30	58.44	1.16	
P65	44.91	61.97	1.21	
P60	48.11	64.21	1.27	
P55	49.88	67.20	1.30	
P50	51.13	71.31	1.33	
P45	53.00	77.76	1.41	
P40	53.82	84.20	1.44	
P35	60.55	93.49	1.48	
P30	64.29	95.08	1.56	
P25	68.32	97.69	1.65	
P20	71.75	102.03	1.75	
P15	76.38	110.89	1.78	
P10	86.14	117.97	1.89	
P5	102.28	161.74	2.33	
P1	121.48	180.27	3.68	

**Table 3:** Means and standard deviations of the vertical time (VT), adjusted horizontal time (AdjHT), ratio, and number of errors for the group with ID, when dividing the sample into two groups according to the percentage of ID, and the group without ID.

	VT (s)	AdjHT (s)	Ratio	Errors
People with ID (*n* = 45)	57.81 ± 21.93	81.68 ± 35.96	1.50 ± 0.59	5.50 ± 8.69
Group 1 (*n* = 25)	47.04 ± 10.48	71.78 ± 32.11	1.55 ± 0.69	4.92 ± 9.24
Group 2 (*n* = 20)	73.37 ± 24.92	98.18 ± 36.99	1.40 ± 0.38	6.47 ± 7.88
People without ID (*n* = 52)	33.58 ± 5.56	35.24 ± 6.68	1.05 ± 0.09	0.00 ± 0.00

## Data Availability

Data are contained within the article.
